# The effect of blue dishware versus white dishware on food intake and eating challenges among residents living with dementia: a crossover trial

**DOI:** 10.1186/s13104-020-05195-y

**Published:** 2020-07-23

**Authors:** Rachael Donnelly, Cindy Wei, Jill Morrison-Koechl, Heather Keller

**Affiliations:** 1 University of Waterloo, 200 University Ave W, Waterloo, ON N2L 3G1 Canada; 2 Schlegel-University of Waterloo Research Institute for Aging, 250 Laurelwood Drive, Waterloo, ON N2J 0E2 Canada

**Keywords:** Food intake, Eating challenges, Dementia, Dining, Blue, Dishware, Physical environment

## Abstract

**Objective:**

Residents living with dementia (RLWD) often experience changes in their visual perception, which could reduce food intake. Inadequate food intake is known to cause malnutrition, which increases the risk of hospitalization, morbidity, and mortality. This study evaluated the effectiveness of using blue dishware compared to white dishware to improve food intake and mitigate eating challenges among 18 RLWD (mean age 84.6 ± 7.9 years, 72.2% female).

**Results:**

A within-within person crossover design determined differences in food intake and eating challenges between blue and white dishware conditions. Five participants responded to the blue dishware and increased their average food intake by ≥ 10%. Responders were not different from non-responders in terms of demographic or health characteristics. The proportion of eating challenges experienced was not significantly different between the blue and white dishware conditions. Percent food intake was significantly greater at lunch (83.5 ± 19.0%) compared to dinner (75.8 ± 22.1%; *p *< 0.0001), regardless of dishware condition. However, there were no significant differences for food intake between the dishware conditions, even after matching food choices. Promoting food intake and reducing eating challenges in RLWD likely needs multi-component interventions targeting meal quality, meal access, and mealtime experience.

*Trial registration* ClincialTrials.gov Identifier: NCT04298788. Retrospectively registered: 6 March 2020, https://clinicaltrials.gov/ct2/show/NCT04298788?term=NCT04298788&draw=2&rank=1.

## Introduction

Approximately 50 million people live with dementia worldwide, with 10 million new diagnoses annually [1]. Dementia is characterized by cognitive impairments [1], including declines in memory, executive function, and communication [2]. Eventually, it becomes difficult for persons with dementia to perform activities of daily living [1–4], and many enter residential care to receive support [3, 5, 6].

Inadequate intake of energy, protein, and micronutrients is common in residential care [7–13], and poor food intake and malnutrition are prevalent among residents living with dementia (RLWD) [7]. An estimated 52% of RLWD are malnourished [7], compared to 19–44% of residents without dementia [7–10]. Malnutrition is associated with hospitalization, impaired quality of life, and increased risk of falls, morbidity, and mortality [3, 12, 14–17].

Disease- and age-related declines in vision can contribute to decreased food intake among RLWD [3, 6, 9, 18–24]. Persons with dementia experience deficits in contrast sensitivity and colour discrimination [19–23]. Thus, insufficient contrast between food items and dishware (e.g., bowls, plates) may reduce food intake [19, 21, 24, 25] and increase eating challenges [9, 17, 18, 21, 24, 25].

Simple, effective interventions that improve food intake among RLWD are desirable. Yet, few studies have investigated how contrast manipulations within the dining room affect resident food intake and eating challenges [19, 25]. Dunne et al. [19] used high-contrast dishware among 9 male residents with Alzheimer’s disease, and found that the enhanced contrast significantly increased food and fluid intake.

Further investigation is needed to corroborate this study, using a larger sample that includes women and attempts to match food choices. The objective of the present study was to determine if blue dishware increases food intake and reduces eating challenges among RLWD in a typical residential care dining room, using a crossover design with matched meals.

## Main text

### Setting and participants

Participants were recruited from a memory care unit within a single retirement home in Southern Ontario, Canada. All unit residents had dementia. Residents were eligible if they could eat independently, and if they typically ate in the dining room or adjoining lounge. The unit coordinator posted study flyers and emailed substitute decision-makers (i.e., family and friends), who were connected with researchers for informed consent. In accordance with the Declaration of Helsinki and the Tri-Council Policy Statement, participants were asked before each meal whether they assented to have their food and dishware weighed [26]. Ethics clearance was provided by the University of Waterloo Office of Research Ethics (ORE#40986).

The unit coordinator collected information from participants’ health records, including age, sex, length of admission on the memory unit (admission to study end date), and current medications. The unit coordinator assessed cognitive status using the Montreal Cognitive Assessment (MoCA; maximum score = 30) [27]; lower scores represent higher cognitive impairment [27].

### Dishware

**Fig. 1:**
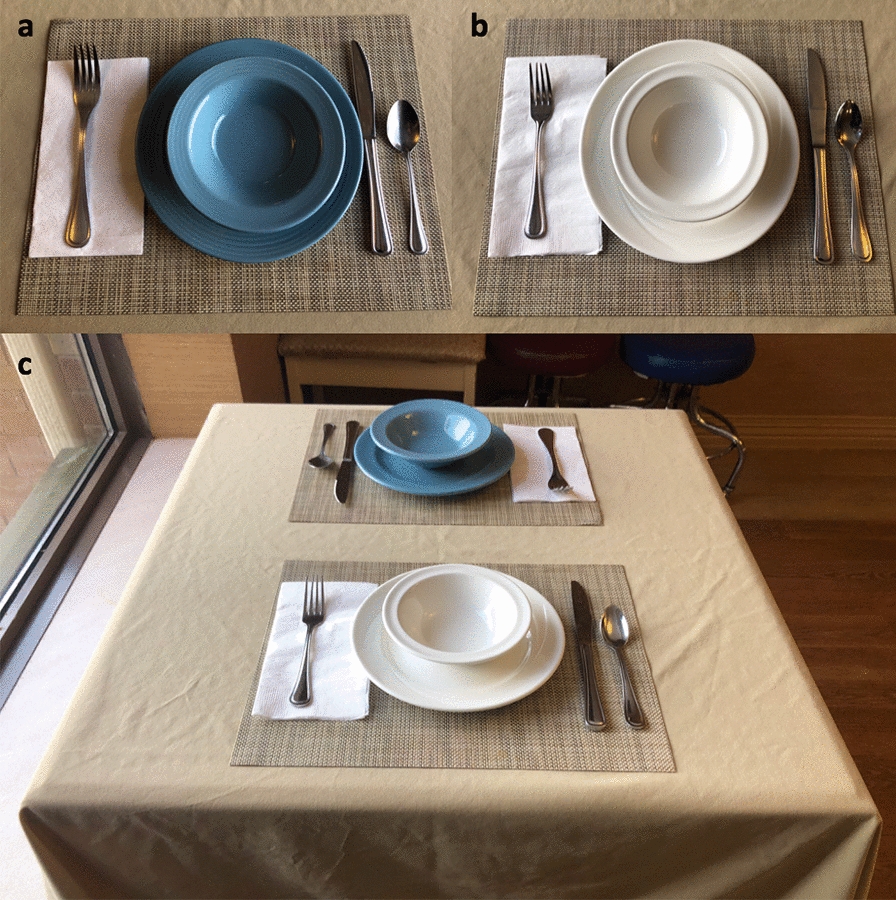
Blue and white dishware on a set table. **a** Blue Lagoon bowl (weight: 411.4 g and inside diameter: 13.6 cm) and plate (weight: 598.3 g and diameter: 23.8 cm) on a beige placemat and tablecloth. **b** Monaco White bowl (weight: 298.9 g and inside diameter: 13.4 cm) and plate (weight: 539.6 g and diameter: 23.8 cm) on a beige placemat and tablecloth. **c** Blue and white dishware set together on a table with a beige placemat and tablecloth.

Blue dishware was ordered from Steelite International© in the shade Blue Lagoon (Fig. 1a), which was the only non-patterned blue dishware commercially available for residential care. White dishware (Manaco White; Fig. 1b) from the same supplier was already present on the unit. Researchers weighed and measured blue and white dishware to calculate average weight. The blue and white dishware were similar in size (blue bowls: 13.6 cm; white bowls: 13.4 cm; blue plates: 23.8 cm; white plates: 23.8 cm; Fig. 1).

### Design

The unit used a 4-week menu cycle. Lunches and dinners were observed for 4 days per week of menu cycle weeks 3 and 4. These weeks had an increased frequency of low-contrast food offerings (e.g., mashed potatoes, white rice). Data collection was completed in 2 rounds. Meals (day of week and menu) were matched to compare blue and white dishware. Dishware conditions were alternated at each meal to reduce the impact of acute illness, dementia progression, or health deterioration on food intake. Data were collected at 32 meals (16 blue, 16 white). The mean number of participant observations was 19 ± 2 meals, with 359 observations in total (178 blue condition, 181 white condition; 176 lunches, and 183 dinners). Participants missed observations due to illness, eating in their room, eating with a loved one, or dissent for observation at a meal. Lunches included soup and a main course, while dinners included only a main course. Meal timing and the number of staff, family, and volunteers present were recorded.

### Food intake

Dishes were labelled with participant numbers, and food and dishware were weighed pre- and post-meal using a ULINE™ Easy-Count food scale accurate to 0.1 grams. After subtracting the average weight of the dishware, the weight of the food consumed was determined by subtracting the leftovers from the food provided. The weight of the food consumed was divided by the weight of the food offered and multiplied by 100 to generate percent food intake.

It was hypothesized that some RLWD may be more likely than others to increase their food intake with blue dishware (i.e., responders). The mean difference in average percent food intake for the dishware conditions was doubled to calculate the threshold value for responders. The threshold value was calculated to be a ≥ 10% increase in food intake with the blue dishware.

### Eating challenges

Participants were observed to determine if eating challenges were present using a standardized checklist [12]: using tableware improperly (e.g., utensils, napkins); spilling food; spitting out food; chewing or swallowing difficulties; falling asleep; or wandering. Eating challenges were recorded as dichotomous variables, and the number of eating challenges observed per participant was totaled for each meal. The total number of eating challenges observed during the study was low. Therefore, the proportion of meals where any eating challenges were observed was compared between the dishware conditions.

### Sample size calculation

A one-way repeated measures within-person analysis of variance (ANOVA) was used to compute required sample size, as there is no current estimation guideline for a two-factor repeated measures within-within design. Based on Dunne et al., the expected effect size was a 25% increase in food intake with the blue dishware. The power and alpha level were set at 80% and 0.05, respectively. At least 16 observations were expected per participant, with a correlation of 0.5 for repeated measurements. The estimated sample size for this study was 11.

### Data analysis

SAS Studio 3.8 (SAS Institute Inc., Cary, North Carolina, United States) was used. Descriptive and bivariate (i.e., Chi square, *t* test) statistics characterized the sample and compared responders to non-responders. A Chi square test compared the proportion of eating challenges experienced during the dishware conditions. A two-factor repeated measures within-within ANOVA compared percent food intake between the dishware conditions and meals. Covariates were: age; sex; MoCA score; length of admission; number of medications; and the number of staff, volunteers, and family present at the meal. A paired t-test compared percent food intake at meals where participants selected the same food choices during matched data collection days. An alpha level of 0.05 was used for all tests.

### Results

Table 1 summarizes the demographic and health characteristics of the sample. Mean MoCA score was consistent with moderate-to-severe dementia [27]. The mean difference in average percent food intake for the entire sample was 4.8 ± 7.0% with the blue dishware. Five participants were classified as responders, but there were no differences between responders and non-responders for demographic or health characteristics. Responders had a significantly greater mean difference in average percent food intake compared to non-responders (*p *= 0.0002; Table 1) for the blue and white conditions. Eating challenges occurred during 48 of the 359 observed meals. Wandering, using tableware improperly, and falling asleep were the most common eating challenges observed. Yet, the number of eating challenges observed was not significantly different between the blue (14.0%) and white (9.4%) dishware conditions (*p *= 0.17).

**Table 1:** Demographic and health characteristics

	All	Responders	Non-responders
Number of residents, *n*	18	5	13
Age, *years*^a^	84.6 ± 7.9	85.0 ± 8.3	84.6 ± 8.5
Sex			
Male, %^b^	27.8 (5)	20.0 (1)	30.8 (4)
Female, %^b^	72.2 (13)	80.0 (4)	69.2 (9)
MoCA score, *points*^a^	12.5 ± 5.0	13.4 ± 3.9	12.3 ± 5.9
Length of admission, *years*^a^	2.3 ± 2.4	1.8 ± 1.2	2.6 ± 2.8
Medications, *#*^a^	9.5 ± 2.5	10.0 ± 1.2	9.2 ± 2.9
Mean difference in average percent food intake, %^a^	4.8 ± 7.0	13.2 ± 2.2^*^	1.6 ± 5.2

^a^Continuous variables are expressed using their mean and standard deviation

^b^Categorical variables are expressed using percentages

^*^Responders had a significantly higher mean difference in average percent food intake compared to non-responders, *p *< 0.05

Table 2 displays mean percent food intake for all meals (unmatched and matched). Percent food intake was significantly greater at lunch compared to dinner (*p *< 0.0001), regardless of dishware condition. When comparing all meals, no significant differences in percent food intake were found for blue or white dishware conditions (*p *= 0.06). Fifteen participants selected the same food choices during 53 matched meals; there was no significant difference in percent food intake (*p *= 0.07).

**Table 2:** Mean percent food intake for all meals and matched meals

	All meals^a^ Mean ± SD	Matched meals^b^ Mean ± SD
Number of residents, *n*	17	15
Number of observations, *n*	332	53
Mean percent food intake at blue meals, *%*	82.1 ± 19.7	87.5 ± 14.8
Mean percent food intake at white meals, *%*	77.1 ± 21.9	83.8 ± 18.4
Mean percent food intake at lunches, *%*	83.5 ± 19.0*	–
Mean percent food intake at dinners, *%*	75.8 ± 22.1	–

^a^The two-way repeated measures ANOVA compared percent food intake between all meals (unmatched and matched). One participant was excluded from this analysis since they were only available for observation during dinners. Other meal observations were excluded from this analysis if covariate data was missing

^b^The paired t-test compared percent food intake between matched blue and white meals for 15 participants

*Percent food intake was significantly higher at lunch compared to dinner, *p *< 0.05

### Discussion

The sample size of this study was double that of Dunne et al., and significant differences in intake were observed between meals (7.7% more food consumed at lunch than dinner). This indicates that the sample was of sufficient size to identify statistically significant differences. However, blue dishware did not significantly improve food intake or mitigate eating challenges compared to white dishware. Most RLWD experienced a non-significant increase of 4.8% for food intake with blue dishware, which is not a clinically meaningful difference. Yet, there was considerable variability, and 27.8% of the sample “responded” to the blue dishware. The average change in food intake for this study is much lower than that found by Dunne et al., who observed a 25% increase in percent food intake with high-contrast blue dishware [19]. This may be due to discrepancies in the shades of blue dishware. However, a direct comparison cannot be made as the shade of blue utilized was not reported by Dunne et al. It is also unclear whether their participants ate in a typical dining room or a controlled environment. The contrast between food and dishware could have been altered due to variance in the presence and colour of table settings (e.g., placemats, tablecloths), and the use of natural versus fluorescent light [25]. Such contrast alterations could have confounded the effect of dishware colour. Thus, the results of the present study have increased external validity as the intervention was completed in a real dining room environment, where researchers did not control for variations in staff, room layout, table setting, or lighting.

Dishware colour also did not reduce eating challenges. RLWD often receive eating assistance to mitigate eating challenges and increase food consumption [11, 16]. Eating assistance facilitates food intake, regardless of dishware colour [12, 17, 28, 29]. Additionally, the ability to eat is one of the last activities of daily living to be lost with dementia [4, 18]. Therefore, the present sample may have experienced too few eating challenges to demonstrate a difference, since those requiring eating assistance were excluded.

Results of this study highlight that the factors impacting food intake among RLWD are complex, and simple interventions may not be sufficient to improve intake. As noted in this study, some residents respond to dishware colour, but the majority did not. Multicomponent interventions tailored to the specific needs of residents are more suitable. The Making the Most of Mealtimes conceptual model and other literature suggest targeting meal quality, access, and experience when addressing the needs and challenges of RLWD [9, 12, 14, 17, 29–34]. This knowledge can be used to develop future studies and meal programs that prevent and treat malnutrition among RLWD in residential care.

## Limitations

The first limitation of this study is the shade of blue dishware used (i.e., Blue Lagoon). This shade may not have produced sufficient contrast with the food, resulting in non-significant findings. Second, although a standardized checklist was used to observe eating challenges [12], some may not have been observed due to the complexity of data collection. Moreover, a validated tool for eating challenges was not used, as the only tool available is the Edinburgh Feeding Evaluation in Dementia scale, which focuses on severe eating challenges associated with eating assistance (e.g., turning head away) [35]. Third, contrast sensitivity and colour discrimination were not assessed. Yet, evidence indicates that visual deficits occur during the early stages of dementia and progress concurrently with the disease [19–22]. Thus, MoCA scores were used as a proxy for this deficit. Fourth, only 53 meals were matched because residents chose different menu options during rounds 1 and 2 of data collection. Last, this intervention was performed in one dining room within a single retirement home. As such, the dining room, serving procedures, and roles of the care staff cannot be considered representative of all memory care units.

## Data Availability

The study protocol and data collection forms are available from the corresponding author upon reasonable request. The datasets generated and analyzed during the current study are not available to researchers outside of the co-investigators due to data protection laws.

## Abbreviations

RLWD: Residents living with dementia
MoCA: Montreal Cognitive Assessment
ANOVA: Analysis of variance
